# American Dental Association

**Published:** 1886-12

**Authors:** 


### AMERICAN DENTAL ASSOCIATION.

TWENTY-SIXTH ANNUAL MEETING, HELD AT NIAGARA FALLS, AUGUST 3D, 4TH, 5TH AND 6TH, 1886.

REPORTED FOR THE INDEPENDENT PRACTITIONER BY "MRS. M. W. J"

(Concluded from page 626.)

#### THURSDAY EVENING SESSION.

After the reading of the minutes and the discussion of some proposed Amendments to Section 1 of Article VI of the Constitution, the work of Section VII was continued.

Dr. H. A. Smith read a paper on

#### THE TONGUE IN HEALTH AND DISEASE.

The tongue being so conspicuously in view during dental operations, its varying appearances, if understood by him, might be made of much benefit to the patient.

Vegetable parasites form the first great source of diseases of the mouth and tongue, furred tongue being regarded by modern science as a growth of fungus, from spores deposited in the papillæ of the tongue. Traumatic ulcers, from the irritation caused by broken teeth, frequently come under the notice of the dentist.

Amalgam fillings are sometimes supposed to cause aphthous patches, but this is probably due to roughness of the fillings or margins, which act as a mechanical irritant. Ragged teeth, rough fillings and ill-fitting plates are enumerated among the causes of cancer of the tongue. The anterior half of the border of the tongue being most frequently affected with the carcinomata, points clearly to the teeth as the offending organs. The slightest lesions of the tongue should be noted; a very slight operation on the part of the dentist may sometimes prevent a simple ulcer from developing into a cancer of the tongue, with its usually fatal termination.

Dr. A. H. Thompson, Topeka, Kansas, read a paper, entitled

#### PATHOLOGICAL HEREDITY AND GOUTY TEETH.

Defects and deformities of the teeth are due to a transmitted predisposition to certain diseases, but we are in the dark as to how this is effected. The pabulum may be perverted by disease, or the formative organ itself may be affected. The action of acid fluids at the time of eruption may affect the enamel, but does not account for defects in the body of the tooth, the former being far less fatal to these organs than the structural injuries due to hereditary influences. The effects of the latter are so invariable as to have a positive diagnostic value to the pathologist. It is very desirable that observations in this direction be made and recorded, particularly by specialists who have access to hospitals and other institutions where large numbers of persons are collected. Gout is not endemic in this country, so that the study of gouty teeth is of less practical importance to American dentists, yet they have a positive diagnostic value in determining the presence of hereditary gout, gouty teeth being recognizable as soon as seen. Their characteristics have been carefully studied by Drs. Duckworth, Carpenter, Stewart, Donkin and others. Milner Fothergill says there is little doubt of their distinctive diagnostic value.

The subjects of Section VII were declared open to discussion.

*Dr. Ingersoll*—Doubted if disease was ever transmitted; only the diathesis or tendency to certain pathological conditions is inherited. A man may have a wart develop at the age of forty years, and his son may have one exactly similar at the same age, but there having been no wart previous to the age of forty it does not come by inheritance, although the tendency existed. The same is true of consumption and other forms of disease said to be hereditary.

*Dr. Morgan*—Thought this distinction a mere play upon words. It is more than a mere tendency which is transmitted. He cited the persistency of the "Morgan" type in horses, their temperament, powers of endurance, gait, etc., running through many years, and the inheritance yet continuing in all horses of that breed. He did not think the diseases of the oral cavity and tongue, referred to by Dr. Smith, fell properly within the province of the dentist. He had never seen but one case where aphthous patches appeared to correspond with amalgam fillings.

*Dr. Brophy*—Thought that incipient cancer was more frequent than we suspected. Many ulcers falling under the observation of the dentist would, if allowed to run on, develop into carcinoma, ending in death, this form of cancer developing very rapidly, and often there would be no such development but for the irritation of a tooth, there being no inherited tendency.

*Dr. Ottofy*—Thought that the condition of the mouth and oral fluids was an important subject for the consideration of the dentist, and hoped that the suggestions of Dr. How would be adopted.

*Dr. Harlan*—Said that the part played by germs in producing acid ferments was now generally recognized. If the germs are destroyed, we no longer have their products, thus correcting one source of acid saliva.

Timely attention by the dentist in removing the cause of incipient lesions may often save the tongue, or a portion of it, or even life itself.

*Dr. Jas. Truman*—Had found that the mixed secretions of the oral cavity were habitually neutral, being acid only at the necks of the teeth and other places at rest. During the night there is but little secretion of alkaline saliva, and at that time the acid secretions predominate. Tests, to be of value, should be made at different hours, both of the day and night.

*Dr. Atkinson*—Congratulated the Association on this discussion of scientific topics, though he regretted the lack of knowledge of the processes of nutrition and of the elements of tissue. Microbes are the antecedents of unhealth. Living bodies die through retrogressive molecular changes, but we don't know the causes; we know only antecedents and effects. Food is churned into pabulum, which is the nutrition of all the tissues carried into needy territories and used for building up the body. Listening, in the mind, is the equivalent of the tissues waiting for pabulum.

*Dr. Thompson*—Said that he did not see the value of the distinction drawn by Dr. Ingersoll between inherited disease and transmitted diathesis. Whether it is the disease or the diathesis that is transmitted, the result is the same when children are born syphilitic. He only wished to bring to notice a certain form of idiosyncrasy.

On motion, the subject was passed. Dr. Crouse offered a report from the Executive Committee, embodying a history of the difficulties encountered in the choice of place of meeting, and recommending several changes in the Constitution, extending the power and authority of the Executive Committee and its Chairman, and asked unanimous consent for the immediate consideration and adoption of the amendments. After a heated discussion, all the personal portions of the report were ordered stricken out, and the report thus amended was accepted.

The proposed Amendments to the Constitution were laid upon the table for one year.

Adjourned to 9 A. M.

FRIDAY MORNING SESSION.

The Association was called to order at the usual hour, the President in the chair. The minutes of the previous session were read and approved.

Drs. L. P. Haskell, W. B. Ames and W. H. Trueman were appointed by the President as a committee to confer with the manufacturers of mineral teeth.

Dr. C. N. Pierce introduced a resolution discountenancing, by the Association, the customary banquets and entertainments, which was unanimously adopted.

A resolution offered by Dr. S. W. Dennis, requesting the different professional and scientific associations of the country to select places and times of the annual meetings of such bodies as nearly uniform as possible, to accommodate members from distant portions of the country, was lost.

A resolution offered by Dr. Marshall, that the Association recommend to the various Dental Colleges that their sessions be uniformly extended to not less than nine months in the year, was referred to the Committee on Education.

By unanimous consent, Section VI of the Constitution was amended as follows: Sections II and III—Dental Education and Dental Literature and Nomenclature—were consolidated as Section II, and a new section, to be known as Section III, Pathology and Surgery, formed.

Asheville, N. C., received a large majority of the votes cast for the next place of meeting.

The list of officers elected has already been published. (See page 529, September issue.) The officers-elect were installed with the usual ceremonies.

The retiring President, Dr. W. C. Barrett, with evident emotion, thanked the members of the Association for their kind consideration. He said that though he had doubtless made mistakes, God knew they were mistakes of the head, not of the heart. It would always be the pride of his life to remember that he had been the President of the American Dental Association.

The President-elect, Dr. W. W. Allport, accepted the chair with brief but well-chosen words. He said that he was neither unmindful of the honor nor ignorant of the responsibilities he was accepting. He hoped that any and all unpleasantness would be forgotten and left with the past, and that their future proceedings would be such as to add to the good name and increase the influence of the profession, and add to that individual reputation which constitutes much of the little we have to leave to our children. He would only promise that he would do all in his power to discharge his duties acceptably.

As his first official act he appointed Dr. E. T. Darby and Dr. A. W. Harlan as Publishing Committee, and Drs. Douglass, Asheville, N. C.; T. T. Moore, Columbia, S. C.; and D. Bland, N. C., a local Committee of Arrangements.

The last items of the final minutes were read and approved, and the Association adjourned to meet at Asheville, N. C., on the first Tuesday in August, 1887.

